# Managing Respiratory Failure in Late Pregnancy

**DOI:** 10.3390/jcm15093449

**Published:** 2026-04-30

**Authors:** Kate Williams, Alastair White, Melanie Nana, Catherine Nelson-Piercy, Luigi Camporota

**Affiliations:** 1 Critical Care Department, Guys and St Thomas’ NHS Trust, London SE1 7EH, UK; kate.williams71@nhs.net (K.W.); alastair.white6@nhs.net (A.W.); 2 Obstetric Medicine Department, Guys and St Thomas’ NHS Trust, London SE1 7EH, UK; melanie.nana@nhs.net (M.N.); catherine.nelson-piercy@nhs.net (C.N.-P.); 3 Department of Women and Children’s Health, Kings College London, London WC2R 2LS, UK; 4 Centre for Human & Applied Physiological Sciences, School of Basic & Medical Biosciences, Kings College, London WC2R 2LS, UK; 5 Division of Anaesthetics, Pain Medicine and Intensive Care (APMIC), Department of Surgery and Cancer, Faculty of Medicine, Imperial College London, London SW7 2AZ, UK; 6 Critical Care Department, Imperial College Healthcare Trust, Hammersmith Hospital, London W12 0HS, UK

**Keywords:** respiratory failure, pregnancy, acute respiratory distress syndrome, maternal critical care, mechanical ventilation, prone positioning, extracorporeal membrane oxygenation, maternal–foetal physiology, obstetric emergencies, hypoxaemia

## Abstract

**Background/Objectives:** Respiratory failure in late pregnancy represents a complex and high-risk clinical scenario due to physiological adaptations during pregnancy that reduce maternal respiratory reserve, with tightly coupled maternal and foetal outcomes. This review aims to synthesise current evidence on epidemiology, maternal–foetal physiology, and management strategies for respiratory failure in late gestation. **Methods:** This narrative review integrates contemporary literature, national surveillance data, physiological principles, and expert consensus to summarise the causes, clinical implications, and management of respiratory failure in pregnancy. **Results:** Respiratory failure in pregnancy arises from diverse obstetric and non-obstetric conditions, including pneumonia, asthma, pulmonary embolism, cardiogenic pulmonary oedema, and ARDS. Maternal hypoxaemia is strongly associated with foetal compromise. Management requires pregnancy-specific ventilatory targets, avoidance of permissive hypercapnia, cautious use of non-invasive and invasive ventilation, and safe implementation of prone or semi-prone positioning. ECMO use has expanded, with maternal survival improving to approximately 75%, although optimal anticoagulation and timing of delivery remain uncertain. **Conclusions:** Effective management of respiratory failure in late pregnancy requires early recognition, multidisciplinary coordination, and adaptation of respiratory support to maternal–foetal physiology. Despite improvements in critical care and ECMO outcomes, key evidence gaps persist, underscoring the need for integrated maternal critical care pathways and further research to optimise outcomes for both mother and baby.

## 1. Introduction

Respiratory failure in late pregnancy represents a unique challenge for critical care clinicians and requires coordinated multidisciplinary management [1,2,3].

Care is complex because of the profound cardio-respiratory, haematological and metabolic adaptations of pregnancy and the early postpartum period [4]. These changes reduce maternal physiological reserve [5] and increase the risk of both maternal decompensation and foetal compromise. Investigations and treatments must also be selected with careful consideration of placental transfer and potential foetal exposure [6].

From a respiratory perspective, functional residual capacity (FRC) decreases by up to 30% as the gravid uterus elevates the diaphragm [5]. Progesterone-mediated hyperventilation increases minute and alveolar ventilation, lowering the PaCO_2_ set point and apnoeic threshold [4]. Cardiac output and oxygen consumption rise by approximately 30% [7]. Although these adaptations maintain adequate gas exchange in health, the combination of reduced FRC, increased oxygen demand and higher intrapulmonary shunt fraction renders pregnant patients more susceptible to hypoxaemia and rapid desaturation during acute illness [3].

Respiratory failure in late pregnancy may result from pregnancy-specific conditions or from pre-existing or intercurrent medical disease, encompassing a broad and heterogeneous range of pathologies. Experience from pandemics such as Influenza A (H1N1) [8] and COVID-19 [9] has shown that pregnant patients may be disproportionately affected and can develop more severe illness [8]. Causes of respiratory failure include pneumonia, asthma, pulmonary embolism, acute respiratory distress syndrome (ARDS), cardiogenic pulmonary oedema, complications of hypertensive disorders of pregnancy, and obstetric emergencies such as amniotic fluid embolism and peripartum cardiomyopathy [9,10,11,12,13,14]. Management priorities focus on maternal stabilisation, while carefully considering foetal viability, uteroplacental perfusion, medication safety, and decisions regarding the timing, mode, and location of delivery.

Critical care teams caring for these patients must account for altered airway anatomy [15], increased aspiration risk, reduced ventilatory reserve and differences in acid–base norms [15]. Adjustments needed in support techniques such as non-invasive and invasive mechanical ventilation, prone positioning [16] and extracorporeal membrane oxygenation (ECMO) must also be considered [17].

This review synthesises current evidence and expert consensus to guide the management of respiratory failure in late pregnancy and the postpartum period.

## 2. Epidemiology of Respiratory Failure in Late Pregnancy

In the United Kingdom, the maternal mortality rate is approximately 12.82 per 100,000 live births, as reported by MBRRACE-UK (Mothers and Babies: Reducing Risk through Audits and Confidential Enquiries) [18]. The UK Obstetric Surveillance System (UKOSS) reports that the rate of severe maternal morbidity is approximately 8–12 per 1000 deliveries [19], while intensive care admission complicates 2.2 per 1000 deliveries based on data from the Intensive Care National Audit & Research Centre (ICNARC) [20]. Respiratory failure, while less common, complicates 1–4 per 10,000 pregnancies, but late pregnancy is associated with an increased risk of respiratory decompensation [4,18,21]. This reflects the higher susceptibility to infections and thromboembolic disease [11], together with reduced physiological reserve [7]. Common causes of respiratory failure in late pregnancy are presented in Table 1.

Asthma is the most common chronic medical condition in pregnancy, affecting 3–12% of women worldwide [11]; with at least one third experiencing worsening symptoms during pregnancy [9]—most commonly between 32 and 34 weeks. Poorly controlled asthma during pregnancy is associated with adverse pregnancy outcomes such as pre-eclampsia, preterm birth, low birth weight, and early life pneumonia and asthma [11].

Pneumonia, particularly viral pneumonia, such as influenza and SARS-CoV-2 infection, remains a leading cause of maternal critical illness [11]. During the recent COVID-19 (SARS-CoV-2) pandemic, pregnant patients were at higher risk of severe disease and acute respiratory distress syndrome, with similar trends as seen with the 2009 H1N1 influenza pandemic and the 2012 Middle East respiratory syndrome coronavirus (MERS-CoV) epidemic [8,9,24].

### Outcomes in Mother and Baby

Maternal mortality in the context of respiratory failure is largely determined by the underlying aetiology and the access to advanced respiratory support, with mortality rates below 5% in well-resourced settings, but may exceed 30% in low-resource environments where access to mechanical ventilation or extracorporeal support is not available [10,17]. As in other critically ill populations, ICU courses may be complicated by ventilator-associated pneumonia, prolonged mechanical ventilation and extended length of stay [20]. In addition, the need for emergency delivery during critical illness further increases maternal morbidity [28].

Foetal outcomes are strongly linked with maternal outcomes; particularly the severity and duration of maternal hypoxaemia [5]. Maternal oxygen saturations below 95% have been associated with impaired foetal oxygen delivery [4]. Adverse consequences include foetal distress, preterm birth and intrauterine growth restriction [18]. Prolonged maternal critical illness also increases the likelihood of iatrogenic prematurity, with its attendant short- and long-term complications [25].

## 3. Cardiorespiratory Physiology in Late Pregnancy

Significant cardiorespiratory physiological changes, mediated by both hormonal and mechanical influences [4], occur to meet the increased metabolic and oxygen demands of pregnancy, ensuring adequate uteroplacental circulation to enable foetal growth [7]. When compromised this can result in both maternal and foetal morbidity. An understanding of these adaptions is important in allowing clinicians to optimise management in women who develop respiratory failure in pregnancy [5].

### 3.1. Implications for Gas Exchange on Maternal and Foetal Health

**Figure 1:**
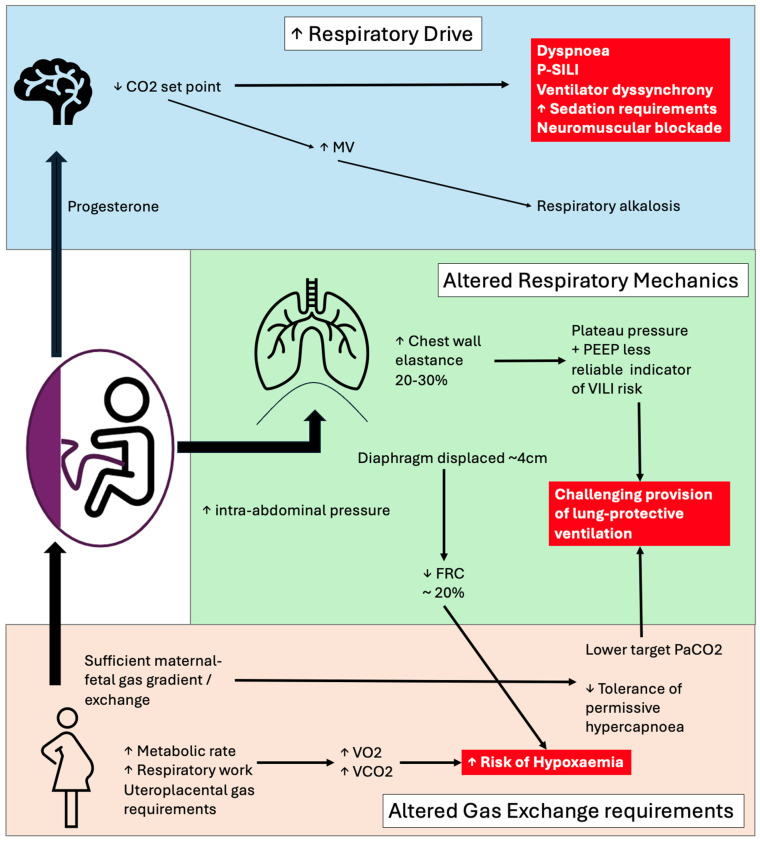
Physiological changes in pregnancy relevant to respiratory failure. Increased respiratory drive, altered respiratory mechanics and gas exchange requirements provide challenges for managing respiratory failure [4,5,31] (P-SILI = patient self-inflicted lung injury; MV = minute ventilation; PEEP = positive end-expiratory pressure; VILI = ventilator-induced lung injury).

**Figure 2:**
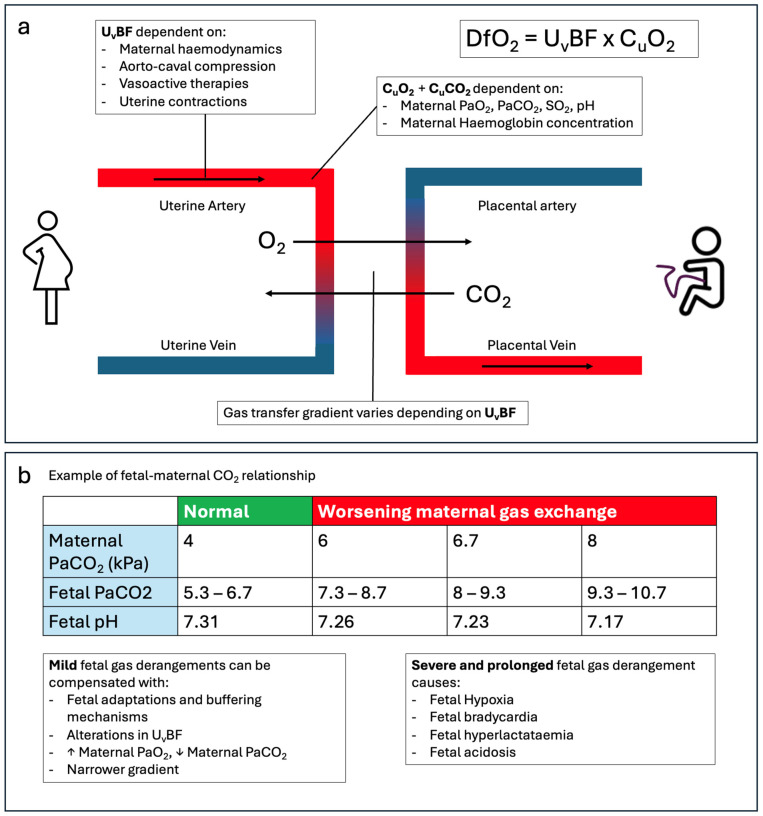
Maternal–foetal gas exchange. (**a**): Delivery of oxygen to the foetus (DfO_2_) depends on uterine arterial blood flow (U_v_BF) and uterine artery blood oxygen content (C_u_O_2_). Optimising the maternal factors which U_v_BF and C_u_O_2_/C_u_CO_2_ are dependent on is needed to maintain sufficient gas transfer gradients to avoid foetal harm. (**b**) Values written as examples of the foetal–maternal CO_2_ relationship are extrapolated physiological estimates and do not represent empirical research [4,5].

Pregnancy is associated with increased maternal oxygen consumption because of the metabolic demands of the developing foetus [7,15]. Progesterone-mediated increases in minute ventilation (Figure 1) result in a compensated respiratory alkalosis and a reduced maternal arterial PaCO_2_, which increases the foetal–maternal carbon dioxide gradient and facilitates placental CO_2_ transfer [4,5]. Foetal oxygen delivery is dependent on adequate maternal oxygenation and uteroplacental perfusion [29] and is therefore highly sensitive to maternal hypoxia and hypotension, as well as vasoconstriction of the placental vessels which can significantly limit maternal–foetal gas exchange and acid–base [29,30] (Figure 2). These changes further reduce the narrow margins for physiological compensation in late pregnancy, with the result that respiratory failure can result in early and severe maternal and foetal compromise.

### 3.2. Haemodynamic Changes

Cardiac output increases in pregnancy. In early gestation, this increase is primarily driven by an expansion in stroke volume, which rises by up to 30% and peaks at approximately 20–24 weeks [32]. As pregnancy progresses, heart rate increases by around 10–20% [33], becoming the dominant contributor to elevated cardiac output in late gestation, with a peak at approximately 34 weeks [34]. Arterial blood pressure falls in pregnancy, reaching a nadir at around 19 weeks’ gestation [35], before gradually increasing to just above pre-pregnancy levels by term [34].

This pattern reflects a marked reduction in systemic vascular resistance, which facilitates adequate uteroplacental perfusion. The fall in vascular resistance activates the renin–angiotensin–aldosterone system, promoting sodium and water retention and leading to progressive plasma volume expansion [36] until approximately 30 weeks’ gestation, thereby supporting circulatory stability [37]. In late pregnancy, the enlarged uterus can compress the inferior vena cava, particularly in the supine position, reducing venous return and cardiac preload [38,39]. In severe respiratory failure, these haemodynamic adaptions further diminish cardiovascular reserve and increase vulnerability to decompensation. Hypoxaemia, sedation, positive pressure ventilation and supine positioning can all precipitate haemodynamic instability, with important consequences for both maternal perfusion and foetal wellbeing.

### 3.3. Mechanics of Ventilation and Hormonal Influences on Respiratory Drive

**Figure 3:**
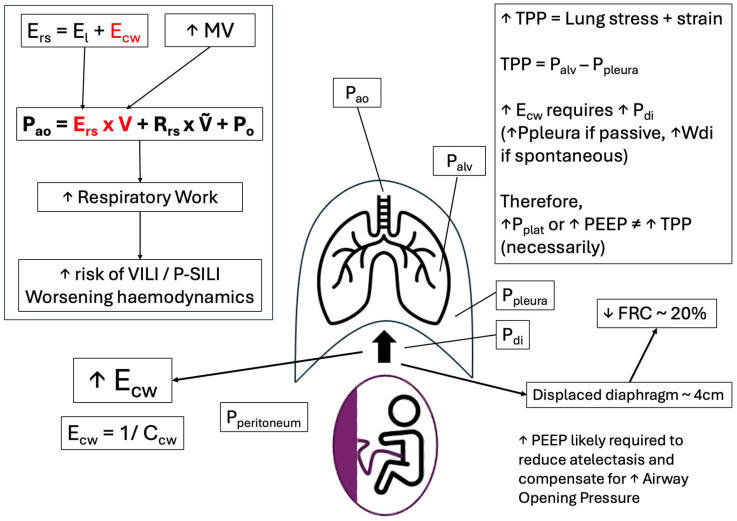
Respiratory mechanics in late pregnancy. While chest wall elastance (E_cw_) is reduced by progesterone-induced ligamentous relaxation, overall, E_cw_ is increased in late pregnancy due to the gravid uterus. Along with the required increase in minute volume (MV), increased respiratory system elastance (E_rs_) necessitates greater applied ventilatory pressures (P_ao_) and therefore respiratory system work. This may increase the risk of ventilator-induced lung injury (VILI) or patient self-induced lung injury (P-SILI). However, increased P_ao_ will only cause alveolar stress and strain, and therefore injury, if the transpulmonary pressure (TPP) is excessive. TPP is the difference between alveolar pressure (P_alv_) and pleural pressure (P_pleura_). Increased intra-abdominal pressure (P_peritoneum_) due to the gravid uterus requires a greater downward force from the diaphragm (Pdi) for inspiration, as well as preventing atelectasis during expiration. Therefore, greater Ppleura is required during mechanical ventilation to overcome increased P_peritoneum_; thus, higher P_alv_, and therefore higher P_ao_, is likely necessary without increasing TPP [3,5,31,41].

Progesterone stimulates central respiratory drive, resulting in increased tidal volume and a chronic physiological increase in minute ventilation, without a significant change in respiratory rate, leading to compensated respiratory alkalosis [4,40]. Oestrogen enhances the effects of progesterone [5], while relaxin, together with progesterone, contributes to increased chest wall compliance through ligamentous relaxation, facilitating thoracic expansion [40,41]. Diaphragmatic elevation and increased intra-abdominal pressure from the gravid uterus result in reduced functional residual capacity, as well as reducing chest wall compliance [5] (Figure 3). The combination of reduced functional residual capacity, increased oxygen consumption and minute ventilation limits the compensatory capacity during acute respiratory illness, predisposing to early and rapid decompensation [3].

### 3.4. Immunological and Hormonal Adaptations Predisposing to Respiratory Failure

Pregnancy is characterised by a coordinated immunological remodelling that supports tolerance of the semi-allogeneic foetus but attenuates maternal defences against specific pathogens. A well-described shift in T-helper cell balance away from Th1 (cell-mediated) towards Th2 (humoral) responses occurs, together with reduced natural killer cell cytotoxicity, attenuated CD8^+^ T-cell activity, and altered macrophage and dendritic cell function [42,43]. These changes are modulated by progesterone, oestrogen and human chorionic gonadotrophin and provide a mechanistic basis for the increased severity of intracellular and viral infections observed in pregnancy, including influenza, varicella-zoster and coronavirus infections [8,9,24,44]. The magnitude of immune adaptation is greatest in the third trimester, coinciding with the period of highest risk of severe maternal respiratory infection.

Sex steroids further modify susceptibility to respiratory failure. Oestrogen-mediated vascular permeability and mucosal hyperaemia produce upper airway oedema, capillary engorgement and reduced nasal patency, contributing to the anticipated difficult airway and increasing the risk of mucosal bleeding during instrumentation [15,45]. The hyperdynamic, hypervolaemic circulation, combined with a reduced plasma colloid osmotic pressure, lowers the hydrostatic threshold for pulmonary oedema, particularly in the context of pre-eclampsia, tocolytic therapy or aggressive fluid resuscitation [46]. Pregnancy also induces a pronounced prothrombotic state, with raised concentrations of factors VII, VIII and X, increased fibrinogen, acquired resistance to activated protein C, and venous stasis from uterine compression; collectively, these predispose to venous thromboembolism and pulmonary embolism, which remains a leading direct cause of maternal mortality [26,27].

Progesterone additionally augments both the slope and the intercept of the ventilatory response to CO_2_, increasing central and peripheral chemosensitivity and producing a chronically elevated minute ventilation at a lower baseline PaCO_2_ (typically 28–32 mmHg in late pregnancy) [4,40,47]. The apnoeic threshold is correspondingly reset to a lower PaCO_2_, narrowing the buffer against transient hypoventilation and contributing to the rapid desaturation observed during induction of anaesthesia, upper airway obstruction or sedation. When the lung becomes injured, this heightened respiratory drive, superimposed on an already elevated ventilatory requirement, becomes maladaptive: vigorous inspiratory effort generates large negative swings in pleural pressure, elevated transpulmonary pressures, regional overdistension and pendelluft, which amplify stress and strain and potentiate patient self-inflicted lung injury (P-SILI) [48,49]. The combination of high metabolic demand, reduced FRC and a high baseline minute ventilation makes non-invasive strategies less effective at unloading the respiratory pump and lowers the threshold at which spontaneous effort becomes injurious. These considerations support close monitoring of inspiratory effort, a low threshold for early invasive ventilation with controlled modes, and, where effort remains excessive, short-term neuromuscular blockade to limit injurious transpulmonary pressures in pregnant patients with progressive hypoxaemic respiratory failure [48]. Taken together with the immunological and endocrine adaptations, these features narrow the physiological margin and help to explain both the increased incidence and the greater severity of respiratory failure observed in late pregnancy.

## 4. Approach to Respiratory Support in Late Pregnancy

Critically ill pregnant patients require close surveillance of both maternal and foetal wellbeing. Standard ICU monitoring should be supplemented by regular foetal assessment, including daily cardiotocography (CTG) and bedside ultrasound where appropriate [50,51]. CTG is only indicated once gestations of foetal viability are reached and if the result of such monitoring would result in urgent delivery. Early and sustained involvement of a multidisciplinary team, obstetric physicians, obstetricians, midwives and intensivists, is essential [50]. Comprehensive maternal monitoring in the critical care setting includes continuous heart rate, invasive or non-invasive blood pressure, oxygenation and haemodynamic assessment to ensure optimal management. Where prolonged mechanical ventilation (>48 h) is anticipated, transfer to a centre with expertise in maternal critical care is recommended (GPICS-2, UK Guidelines for the Provision of Intensive Care Services) [50].

During inter-hospital transfer, careful attention must be paid to maternal positioning to minimise aortocaval compression, typically through left lateral tilt [38,39]. Teams should prepare for unexpected events en route, including the possibility of delivery, with appropriate personnel, equipment and medications immediately available [51].

**Figure 4:**
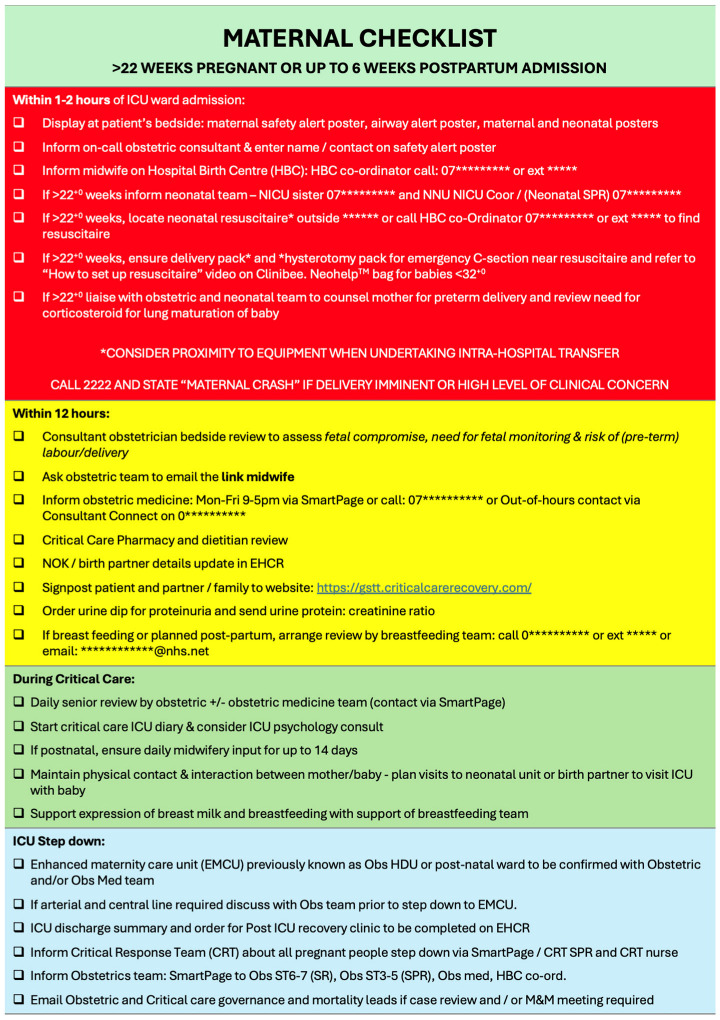
Example of maternal checklist.

Given the relatively low incidence of maternal critical care admissions, staff familiarity may be variable. A structured bedside checklist can support safe practice, including clear escalation pathways, maternal cardiac arrest protocols and key contact details [14] (see Figure 4). In the event of maternal cardiorespiratory arrest, perimortem caesarean section (resuscitative hysterotomy) should be undertaken within 4 min [52]. A predefined plan, with readily accessible equipment and drugs, is therefore essential.

Obstetric patients may require ventilation for a wide range of indications that can lead to severe respiratory failure, just like the general population. Physiological changes in pregnancy and the need to consider both maternal and foetal wellbeing present unique challenges in their management [1,3] and, because of this, careful assessment of the ventilatory strategies used including prone and semi-prone positioning are needed to optimise respiratory function whilst minimising potential risks [53,54].

### 4.1. Non-Invasive Respiratory Support

A tiered approach to oxygenation strategies is used in ARDS in pregnancy [55]. Non-invasive ventilation (NIV) is effective for obstetric patients with mild–moderate ARDS and has been shown to be superior to high-flow nasal oxygen (HFNO) [56]. The risk of gastric aspiration is increased in these patients due to reduced lower oesophageal sphincter tone and increased gastric pressure from the gravid uterus [15], so NIV should be used with caution, in awake and alert patients in upright position with early escalation if no improvement/deterioration.

Awake proning, either by themselves or with assistance, is recommended if able [16], with both semi-prone and full proning possible so long as appropriate support is in place (such as pillows) to support the gravid uterus and allow ongoing assessment of the foetus [54,56]. These patients are at risk of deterioration and so a clear de-proning plan with emergency airway equipment should be readily available at the bedside [16].

### 4.2. Invasive Mechanical Ventilation

A difficult intubation can be more likely with obstetric patients due to both anatomical and physiological changes. A combination of laryngeal oedema, increased BMI and increased breast size can cause issues with intubation. An experienced airway clinician should be involved [15], along with optimal positioning (ramp, left lateral tilt/manual uterine displacement to avoid aortocaval compression), adjusted equipment (including a short-handled laryngoscope/video laryngoscopy) and use of the OAA-DAS guidelines/checklist (Obstetric Anaesthetic Association and Difficult Airway Society) [14], including a plan for failure to intubate and oxygenate the patient to increase success and reduce the risk of adverse events.

Pregnant patients have a higher baseline minute ventilation to facilitate placental gas exchange [5], and their ventilatory targets therefore differ from those used in the general ICU population. In established respiratory failure, maternal PaO_2_ is typically maintained above 9 kPa, and PaCO_2_ is targeted within the physiological pregnancy range of approximately 3.7–4.3 kPa. Hypercapnia may result in foetal acidosis, while excessive hypocapnia can reduce uteroplacental blood flow [4]; consequently, permissive hypercapnia is generally avoided in this population.

Lung-protective ventilation remains the cornerstone of management, with tidal volumes of approximately 6 mL/kg predicted body weight, plateau pressures < 30 cmH_2_O, driving pressures < 15 cmH_2_O, and appropriate levels of positive end-expiratory pressure (PEEP) [55,57]. However, strict adherence to lung-protective parameters may result in relative hypoventilation when compared with the higher physiological minute ventilation of pregnancy [8], which is largely achieved through increased tidal volume in health. Notably, pregnant patients were excluded from the landmark trials underpinning this strategy.

Increased chest wall elastance in late pregnancy may further influence airway pressures [5,41]. Measurement of transpulmonary pressure using an oesophageal balloon may therefore help to guide safe adjustments in ventilation [58], allowing optimisation of minute ventilation while minimising the risk of ventilator-induced lung injury (Figure 3). PEEP must be carefully titrated, balancing alveolar recruitment against the risk of elevated intrathoracic pressures, reduced venous return and subsequent maternal haemodynamic compromise, with potential downstream effects on uteroplacental perfusion [59].

Airway pressure release ventilation (APRV) remains a controversial mode of mechanical ventilation but has theoretical benefits of maximising alveolar recruitment while minimising alveolar stress [60]. While its safe use for refractory hypoxaemia in pregnant patients has been reported [61], we would recommend doing so only with the assurance of regular foetal monitoring.

Neuromuscular blockade is commonly used in the management of the intubated non-obstetric population with severe respiratory failure to reduce patient–ventilator dyssynchrony and oxygen demand [62]. While comprehensive data to fully inform maternal and foetal risk are lacking, placental transfer is minimal [63] and utilisation of neuromuscular blockade in severe respiratory failure in late pregnancy is well documented [10].

Improvement in oxygenation and ventilation with the use of prone positioning leading to improved ventilation-perfusion matching, reduced shunting and consequent lung recruitment is an established strategy for management of severe respiratory failure [64]. It can be used in the obstetric population and has been proven to be safe even in late gestation; the use of pelvic and chest bolsters to accommodate the gravid uterus can avoid uterine compression [16]. Both maternal and foetal monitoring should continue in this position, to detect evidence of foetal distress. Semi-prone positioning is an alternative in those patients in whom prone positioning is not tolerated [54,65].

Inhaled nitric oxide can improve gas exchange in ARDS but without evidence of a mortality benefit [66]. Its safe use in pregnancy is described in only a few case series [67], and therefore it can only be recommended as a short-term bridge to another therapy such as ECMO.

### 4.3. Extracorporeal Support

ECMO is an advanced life support technique that is used in cases of respiratory and cardiovascular failure when conventional intensive care therapies have failed. It has been used to support pregnant and postpartum women presenting with several cardiac and respiratory pathologies [17], increasingly following the COVID-19 pandemic [10]. MBRRACE has highlighted the importance of early referral to an ECMO-capable centre to improve outcomes for parturients with significant respiratory failure [18].

Respiratory indications for veno-venous membrane oxygenation (V-V ECMO) initiation in the obstetric population include severe acute respiratory distress syndrome [17] often secondary to viral pneumonia, (influenza, COVID-19, or H1N1) [8,9,24]; bacterial pneumonia, aspiration, trauma, and inflammatory lung diseases; and massive pulmonary embolism leading to severe hypoxaemia and right heart failure and asthma [17,68,69]. ARDS remains the most common respiratory indication, with viral pneumonia being the most frequent cause.

Close monitoring of both mother and foetus is necessary once ECMO has commenced using regular ultrasounds and CTGs once foetal viability has been reached and only if urgent delivery in the event of CTG concerns is deemed appropriate. Specific considerations in obstetric ECMO patients are in Table 2.

In hospital, survival for pregnant and postpartum patients requiring ECMO has significantly improved from 46% to 75% in recent years [17,25]. Foetal survival is dependent on gestational age and maternal stability/severity of illness but is around 70–75% [25]. Early referral to a specialist ECMO centre for early cannulation, with co-located specialist obstetric and obstetric medical support, is key to improved outcomes [17,18].

## 5. Areas of Uncertainty and Future Developments

Despite advances in maternal critical care, there are still several uncertainties in the management of severe respiratory failure during pregnancy. One such area is the timing of delivery; although delivery reduces oxygen consumption and diaphragmatic pressure, only small gains in maternal oxygenation have been seen in previously reported studies [68,69]. Delivery therefore should only be carried out after MDT discussions for obstetric indications, not primarily to improve respiratory dynamics [2,28].

Although HFNO, NIV and awake proning were used during the COVID-19 pandemic, there are limited randomised data in pregnant populations [9] and the potential risk of delayed intubation needs to be considered [16,53,56,57].

Ventilatory targets for pregnant patients are another area of uncertainty. Conventional ARDS management advocates for permissive hypercapnia [55]; however, this is poorly tolerated by the foetus and can lead to foetal acidosis [29,30]. Future studies looking at foetal neurological outcomes following episodes of maternal hypercapnia, and in turn looking to define the safe upper limits of maternal pCO_2_, would be of interest [2,69].

We have seen significant improvements in maternal survival with ECMO; however, optimal anticoagulation regimes and timing of delivery whilst on ECMO remain unclear [10,19], as well as longer-term neurodevelopmental outcomes for the surviving infants [74,75]. There is wide variability in access and outcomes given there are few centres that have the necessary expertise and MDT support for maternal ECMO.

Future maternal critical care systems require integrated pathways that coordinate obstetric, obstetric medicine, critical care, neonatology and retrieval teams [68]. Enhanced early warning tools such as the new Maternity Early Warning Score implemented in the NHS (which gives targeted parameters to use in late pregnancy) [75,76], alongside standardisation of maternal critical care guidelines, should improve outcomes.

## 6. Conclusions

**Figure 5:**
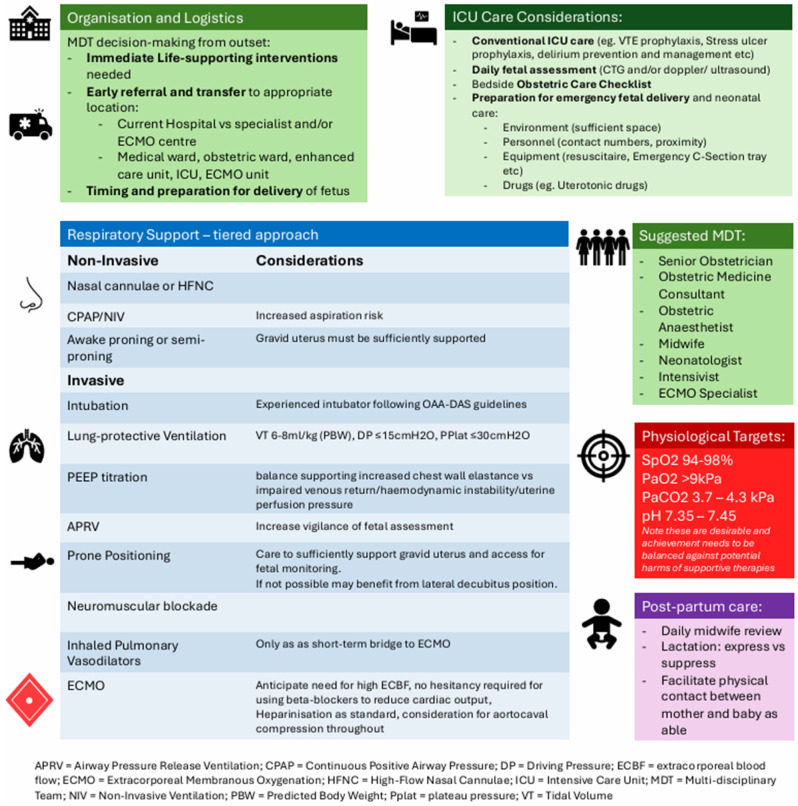
Recommendations for the management of respiratory failure in late pregnancy [9,19,50,54,56].

Respiratory failure in late pregnancy is a high-risk clinical scenario driven by profound physiological adaptations that reduce maternal reserve and tightly link maternal and foetal outcomes. A wide range of obstetric and non-obstetric pathologies can precipitate rapid decompensation, with maternal hypoxaemia and haemodynamic instability directly compromising foetal wellbeing. Optimal management prioritises maternal stabilisation while accounting for altered respiratory mechanics, haemodynamic vulnerability, and the need for pregnancy-specific ventilatory and gas-exchange targets (Figure 5). Successful care relies on early recognition, multidisciplinary collaboration, and access to advanced respiratory support, including prone positioning and ECMO in selected cases. Ongoing uncertainties around ventilatory targets, timing of delivery, and ECMO management highlight the need for further research and the development of integrated maternal critical care pathways to continue improving outcomes for both mother and baby.

## Figures and Tables

**Table 1:** Common causes of respiratory failure in late pregnancy.

	Incidence in Pregnancy	Initial Management	Key Considerations
Severe/life-threatening asthma exacerbation requiring hospital admission [9]	6% of woman with prenatal asthma	- Short-acting beta-2 agonists - Muscarinic antagonists - Systemic corticosteroids - IV Magnesium - Methylxanthines	More common in 2nd and 3rd trimester
Pulmonary oedema [11,12,13,22]	0.08% to 0.5% of pregnancies; common complication of peripartum/dilated cardiomyopathy, complicates 2.9% of pre-eclampsia	- Reduce left ventricular (LV) afterload if hypertensive - Reduce LV preload with diuretics - Treat the cause	Most commonly occurs with fluid dynamics during peripartum period. May be complication of tocolytic or uterotonic therapy
Bacterial pneumonia [11,23]	Incidence not higher but greater morbidity and mortality than general population	- Protocol-guided antibiotics and supportive care	Higher risk of maternal and foetal complications Avoid tetracyclines and quinolones
Viral pneumonia [8,9,24]	Incidence not higher but greater morbidity and mortality than general population	- Disease-dependent - Supportive therapy	Significantly greater risk of severe disease and mortality seen in pregnant patients compared to general population in Influenza and coronavirus pandemics
ARDS [10,25]	15.9 to 130 per 100,000 deliveries	- Treat the cause - Conventional supportive therapies - Avoid dexamethasone (except for foetal lung maturation) for immunomodulation due to risk to foetal neurodevelopment	Causes include: - Pneumonia - Aspiration - Sepsis (pyelonephritis, chorioamnitis) - TRALI (transfusion-related acute lung injury) - Pancreatitis
Pulmonary embolism [26,27]	1.3 per 10,000 pregnancies	- Systemic or catheter directed thrombolysis if haemodynamically unstable - Treatment dose anticoagulation if haemodynamically stable	If haemodynamically stable, for diagnosis consider performing lower limb ultrasound assessment as if evidence of DVT then treatment is indicated without need for ionising radiography

**Table 2:** Specific considerations in obstetric ECMO patients.

	General Population	Obstetric Patients
**Cardiovascular** Cardiac output [10,19,32,33]	4–8 L/min at baseline Usual initial ECMO flows 3–4 L/min	Due to higher heart rate and stroke volume, increased 6–10 L/min at baseline Usual initial flows between 5 and 6 L/min To reduce recirculation complication may benefit from beta-blockade to reduce metabolic demand
**Pulmonary/acid-base** PaO_2_ pH PaCO_2_ [4,5,17]	Usually maintained >8 kPa Usually target >7.30 Usually target 4.5–6 kPa unless right heart dysfunction; can tolerate permissive hypercapnia	Higher target of >9.3 kPa Required for foetal oxygenation Maternal acidaemia poorly tolerated by foetus; therefore, normal pH targeted ~7.40 Maternal hyper- or hypocapnia associated with foetal hypoxemia and acidosis and so more tightly controlled (target 3.7–4.3 kPa)
**Haematological** [18,70,71]		Pregnancy is a hypercoagulable state Unfractionated heparin is used as standard in ECMO and safe in obstetric population (does not cross placenta and not associated with congenital abnormalities) Increased bleeding risk around delivery/postpartum
**Cannulation** [15,19,72,73,74]		Aortocaval compression may cause issues—left uterine displacement may ease guidewire placement (with wedge or pillow under R hip) Foetal monitoring during cannulation (heart rate) to aid in identifying altered uterine blood flow during cannulation. Jugular cannulation may be considered.
**Positioning** [16,38,39,54,73]		Aortocaval compression must be considered throughout Left uterine displacement when supine Proning requires extra considerations and adequate protection/support around gravid uterus
**Delivery** [14,28,50]		Timing and mode of delivery require careful planning, and a multidisciplinary approach is necessary including obstetricians, obstetric medicine team, neonatologists, and obstetric anaesthesia, as well as critical care team.

## Data Availability

No new data were created or analyzed in this study.

## Abbreviations

The following abbreviations are used in this manuscript:

ARDS	Acute respiratory distress syndrome
ECMO	Extracorporeal membrane oxygenation
FRC	Functional residual capacity
TRALI	Transfusion-related lung injury
P-SILI	Patient self-inflicted lung injury
MV	Minute ventilation
PEEP	Positive end-expiratory pressure
VILI	Ventilator-induced lung injury
VO_2_	Volume of oxygen consumption
VCO_2_	Volume of carbon dioxide production
DfO_2_	Delivery of oxygen to the foetus
U_v_BF	Uterine arterial blood flow
C_u_O_2_	Uterine artery blood oxygen content
E_cw_	Chest wall elastance
E_rs_	Respiratory system elastance
E_l_	Lung elastance
R_rs_	Respiratory system resistance
P_ao_	Applied ventilatory pressures
C_cw_	Chest wall compliance
TPP	Transpulmonary pressure
P_alv_	Alveolar pressure
P_pleura_	Pleural pressure
P_peritoneum_	Intra-abdominal pressure
P_di_	Diaphragmatic pressure
W_di_	Diaphragmatic work
CTG	Cardiotocography
NIV	Non-invasive ventilation
HFNO	High-flow nasal oxygen
APRV	Airway pressure release ventilation
